# Genomic Decoding of *Bacillus velezensis* BV01 Reveals Multifaceted Biocontrol Mechanisms Against Fungal Phytopathogens

**DOI:** 10.3390/microorganisms14102285

**Published:** 2026-10-08

**Authors:** Xiao-Qian Wu, Wen-Ying Zhuang, Zhao-Qing Zeng

**Affiliations:** 1 State Key Laboratory of Microbial Diversity and Innovative Utilization, Institute of Microbiology, Chinese Academy of Sciences, Beijing 100101, China; wuxq1030@foxmail.com (X.-Q.W.); zhuangwy@im.ac.cn (W.-Y.Z.); 2 University of Chinese Academy of Sciences, Beijing 100049, China

**Keywords:** antifungal activity, biocontrol, fungal phytopathogens, genomic analysis, volatile organic compounds

## Abstract

Plant disease biocontrol represents an effective and ecologically sustainable approach for managing fungal phytopathogens. Although our previous study confirmed that *Bacillus velezensis* BV01 possesses potent antagonistic activities against phytopathogenic fungi, the dose-dependent efficacy of its fermentation broth, the contribution of volatile metabolites, and the genetic determinants underlying this inhibition have remained unclear. In this study, strain BV01 markedly suppressed the mycelial growth of *Botrytis cinerea*, *Fusarium graminearum*, *F. oxysporum*, and *Rhizoctonia solani*. Moreover, its volatile organic compounds exerted significant inhibitory effects on these pathogens, albeit to a lesser extent than the fermentation broth. Genomic analyses identified 15 secondary metabolite biosynthetic gene clusters, including those involved in the biosynthesis of macrolactin H, bacillene, bacillibactin, and bacilysin, as well as numerous genes encoding lytic enzymes. Collectively, these findings underscore the considerable potential of *B. velezensis* BV01 as a biocontrol agent and provide a robust foundation for its future development and practical deployment.

## 1. Introduction

Fungal phytopathogens cause diseases that severely compromise plant growth, resulting in substantial yield and quality losses across many major crops and posing a persistent threat to agricultural production and global food security [1]. Moreover, many of these pathogens are capable of releasing millions of spores, inflicting recurrent pre- and post-harvest damage and thereby compounding their overall impact on agricultural productivity [2]. According to statistics from the Food and Agriculture Organization of the United Nations, the five most important global food crops (rice, wheat, maize, soybean, and potato) are all threatened by various fungal diseases [3,4], highlighting the urgent need for an effective control strategy. Currently, the management of fungal phytopathogens predominantly relies on chemical fungicides [5,6]. Although these chemicals can effectively suppress plant diseases in the short term, their prolonged applications not only induce pathogen resistance but also lead to environmental contamination, thereby threatening ecological security [7,8,9]. In contrast, microbial-based biocontrol approaches offer notable advantages in terms of environmental safety and target specificity and are emerging as a promising strategy for the management of plant fungal diseases [10,11]. These microbes can suppress fungal phytopathogens through diverse mechanisms, including competition for nutrients, mycoparasitism, and antibiosis [12,13]. Among these mechanisms, the production of antimicrobial secondary metabolites plays a vital role in biocontrol activity, as these compounds can directly antagonize fungal pathogens and thereby facilitate plant root colonization [14,15]. Therefore, exploring the biosynthetic potential of biocontrol strains is essential for understanding their antagonistic mechanisms and developing more effective biocontrol agents.

A wide array of biocontrol microorganisms has been reported to control plant pathogens, including species from the genera *Bacillus*, *Pseudomonas*, *Streptomyces*, *Trichoderma*, and *Saccharomyces* [16,17,18,19,20]. Among these, many *Bacillus* strains attract considerable interest for the biocontrol of fungal phytopathogens, owing to their strong reproductive capacity, remarkable stress resistance, and multifaceted antagonistic mechanisms [21,22]. These strains are known to produce various antimicrobial metabolites, including lipopeptides, polyketides, lytic enzymes, and volatile organic compounds (VOCs), which exhibit a broad spectrum of inhibitory activity against phytopathogenic fungi [23,24,25,26]. For example, *B. velezensis* strain BZR 517 can produce iturin and surfactin, which disrupt the mycelium of *Fusarium graminearum*, causing leakage of nucleic acids and proteins and thereby inhibiting its growth [27]. *Bacillus velezensis* strain N23 demonstrated significant antagonistic activity against nine phytopathogenic fungi, and contains genes involved in the biosynthesis of antifungal metabolites, including fengycin, surfactin, and iturin [28]. *Bacillus velezensis* strain LW-66 exhibited broad-spectrum antifungal activity against many pathogens, and its VOCs significantly inhibited the growth of *Valsa mali* [29]. *Bacillus velezensis* strain CE 100 produced chitinases and β-1,3-glucanases, which help degrade the cell walls of pathogenic fungi such as *Macrophomina phaseolina* and *F. oxysporum*, thereby effectively inhibiting mycelial growth and controlling strawberry diseases [30]. To date, *Bacillus* species such as *B. velezensis*, *B. subtilis*, and *B. amyloliquefaciens* are well documented as effective biocontrol agents in agriculture settings [22,31].

Whole-genome sequencing, a high-throughput technology, can rapidly acquire complete genomic information for microorganisms and has been widely applied in studies of biocontrol *Bacillus* species. In recent years, whole-genome sequencing has been performed for numerous biocontrol strains, including *B. velezensis*, *B. amyloliquefaciens*, *B. subtilis*, *B. siamensis*, *B. atrophaeus*, and *B. pumilus* [32,33,34]. Whole-genome analysis has shown that these species generally carry multiple gene clusters for the synthesis of antimicrobial compounds, which constitute an important genetic basis for their antagonism against pathogens [28,29,30,32,33,34]. For example, about 10% of the genomic resources of *B. velezensis* strain FZB42 are devoted to the synthesis of various secondary metabolites [35]. Whole-genome sequencing of these microbes contributes to a systematic understanding of their multiple mechanisms and provides a theoretical basis for the development and application of biocontrol agents.

Our previous work demonstrated that *B. velezensis* BV01 exhibits strong antagonistic activity against eight phytopathogens [36]. However, the concentration-dependent antifungal efficacy of its fermentation broth, inhibitory effects of its volatile metabolites, and underlying genetic basis of the antagonism remain unclear. In this study, we aimed to evaluate its antagonistic activities in fermentation broths at different concentrations and volatile metabolites produced by strain BV01, and to identify functional genes associated with phytopathogen suppression through whole-genome sequencing. The findings will provide a theoretical foundation for functional characterization of BV01 as a biocontrol agent and further support its potential applications in agriculture.

## 2. Materials and Methods

### 2.1. Strains and Growth Conditions

Strain BV01 was cultured at 28 °C on Luria–Bertani (LB) medium containing 10.0 g/L peptone, 5.0 g/L yeast extract, 10.0 g/L NaCl for broth, and supplemented with 20.0 g/L agar for solid plates. The fungal pathogens *Botrytis cinerea* PP2, *Fusarium graminearum* PP18, *F. oxysporum* F1, and *Rhizoctonia solani* PP11 were grown on potato dextrose agar (PDA, 200 g/L potato, 20.0 g/L glucose, 20.0 g/L agar) at 25 °C. All strains were preserved in the State Key Laboratory of Microbial Diversity and Innovative Utilization, Institute of Microbiology, Chinese Academy of Sciences.

### 2.2. Antifungal Activity In Vitro

To determine the inhibition effect of VOCs produced by strain BV01 on pathogen growth, a mycelial plug (5.0 mm in diameter) taken from the actively growing colony margin of each pathogen was placed at the center of a PDA plate. They were then inverted and placed onto LB agar plates pre-inoculated with 100 µL of BV01 fermentation suspension (1 × 10^5^ CFU/mL) [37]. The LB plates inoculated with only sterile double-distilled water (ddH_2_O) served as the control. All plates were incubated at 25 °C for 2–4 days, and then the inhibition rates were calculated to assess the antagonistic effects of BV01-derived VOCs against the target pathogens.

To measure the inhibitory effect of cell-free culture filtrate of strain BV01 on the mycelial growth of the pathogens, 100 µL of bacterial suspension (1 × 10^7^ CFU/mL) was inoculated into 100 mL of liquid LB medium and incubated at 28 °C on a rotary shaker at 180 rpm for 2 days. The culture was then centrifuged at 8000 rpm for 10 min, and the supernatant was filtered through a 0.22 µm filter to obtain the sterile filtrate. The filtrate was subsequently mixed with molten PDA (45–50 °C) to reach final concentrations of 1%, 5%, 10%, and 20% (*v*/*v*). PDA plates supplemented with equivalent volumes of sterile liquid LB medium served as control. A 5.0 mm mycelial plug of each pathogen was placed at the center of each PDA plate. All plates were incubated at 25 °C for 3–4 days. The colony diameter of each pathogen was measured.

All treatments were performed in triplicate. The mycelial growth inhibition rate was calculated using the following formula: inhibition rate (%) = [(D_c − D_t)/D_c] × 100%, where D_c is the average colony diameter in the control group, and D_t is the average colony diameter of the treatment group.

### 2.3. Genome Sequencing, Assembly, and Annotation of BV01

A single colony of BV01 was picked and inoculated into LB liquid medium, followed by overnight incubation at 28 °C with shaking at 180 r/min. The resulting culture was centrifuged at 8000 r/min for 10 min at 4 °C to harvest the cells. The cell pellet was then washed twice with phosphate buffer (pH 7.0) and collected for subsequent DNA extraction. The genomic deoxyribonucleic acid (DNA) of strain BV01 was extracted using a modified cetyltrimethylammonium bromide (CTAB) protocol, with its concentration and purity assessed using a Qubit Fluorometer (Invitrogen, Carlsbad, CA, USA) and a NanoDrop Spectrophotometer (Thermo Scientific, Wilmington, DE, USA), respectively.

The whole-genome sequencing was performed on the Illumina Novaseq platform by Personal Biotechnology Company (Shanghai, China). Sequencing libraries were constructed with the TruSeq DNA Sample Preparation Kit (Illumina, SanDiego, CA, USA). Raw reads were trimmed of adapter sequences and filtered low-quality bases using fastp v0.23.1 [38]. The cleaned reads were subsequently assembled into contigs and scaffolds by SPAdes v3.15.4 [39], genome quality assessment was performed through CheckM v1 [40], and the final assembly was polished using Pilon v1.24 [41].

The genome circle plot was generated using Circos v0.69-3 [42]. Protein-coding genes were predicted by GeneMarkS v4.32 [43]. Non-coding RNAs were identified as follows: tRNAs via tRNAscan-SE v2.0.10 [44], rRNAs through Barrnap v0.9 (https://github.com/tseemann/barrnap, accessed on 18 April 2025), and other ncRNAs by searching against the Rfam v12.2 database [45]. Protein-coding genes were functionally annotated using eggnog-mapper v2.1.12 [46], which assigned sequences to eggNOG (COG) categories. The assignments were then summarized across the 26 functional classes [47]. Based on the Gene Ontology (GO) IDs predicted by eggnog-mapper, GO Slim annotation was performed with map2slim and the Generic file [48,49]. KEGG Orthology (KO) and Pathway annotations were performed using the KEGG KAAS automated annotation system (https://www.genome.jp/kegg/, accessed on 18 April 2025). The “For Prokaryotes” gene set was selected, with the annotation method set to “BLAST” and the bidirectional best hit (BBH) rule applied for KO assignment. After KO annotation, KOs were mapped to corresponding KEGG pathways for statistical analysis [50]. Carbohydrate-active enzymes were predicted by Carbohydrate-Active Enzymes v12 (CAZy) database [51], and secondary metabolite biosynthetic gene clusters were identified with the AntiSMASH v7.1.0 [52].

### 2.4. Taxonomic Identification of BV01 Based on ANI and DDH Analysis

To determine the taxonomic position of strain BV01, average nucleotide identity (ANI) and DNA-DNA hybridization (DDH) analyses were performed against ten other *Bacillus* strains. The genome sequences of these strains were retrieved from the National Center for Biotechnology Information (NCBI) database, and their NCBI RefSeq accession numbers are listed in Table 1. The ANI values were computed using the JSpeciesWS (http://jspecies.ribohost.com/jspeciesws/, accessed on 27 April 2025) [53], while DDH analysis was conducted with the Genome-to-Genome Distance Calculator (https://ggdc.dsmz.de/, accessed on 27 April 2025) [54]. An ANI value of ≥95% together with a DDH value of ≥70% served as the criteria for delineating species [53,54].

### 2.5. Comparative Genomic Analysis of BV01

For comparative genomics analysis of BV01, four well-characterized *Bacillus* strains, *B. amyloliquefaciens* DSM7, *B. velezensis* FZB42 and SQR9, and *B. subtilis* 168, served as reference strains. Their genome sequences were downloaded from the NCBI database (https://ncbi.nlm.nih.gov/datasets/genome/, accessed on 26 April 2025). Pan-genome analyses were performed using BPGA v1.3 with default parameters (50% sequence identity cutoff, USEARCH clustering) [55]. A Venn diagram was generated using jvenn (http://jvenn.toulouse.inra.fr/, accessed on 6 June 2025) to visualize shared and unique gene sets among the five strains.

### 2.6. Statistical Analyses

All statistical analyses were performed using SPSS Statistics 20 (IBM Corp., Armonk, NY, USA). Significant differences among treatments were assessed by one-way analysis of variance (ANOVA) followed by Duncan’s multiple range test, with statistical significance set at *p* < 0.05.

## 3. Results

### 3.1. Antifungal Activity of BV01 In Vitro

**Figure 1:**
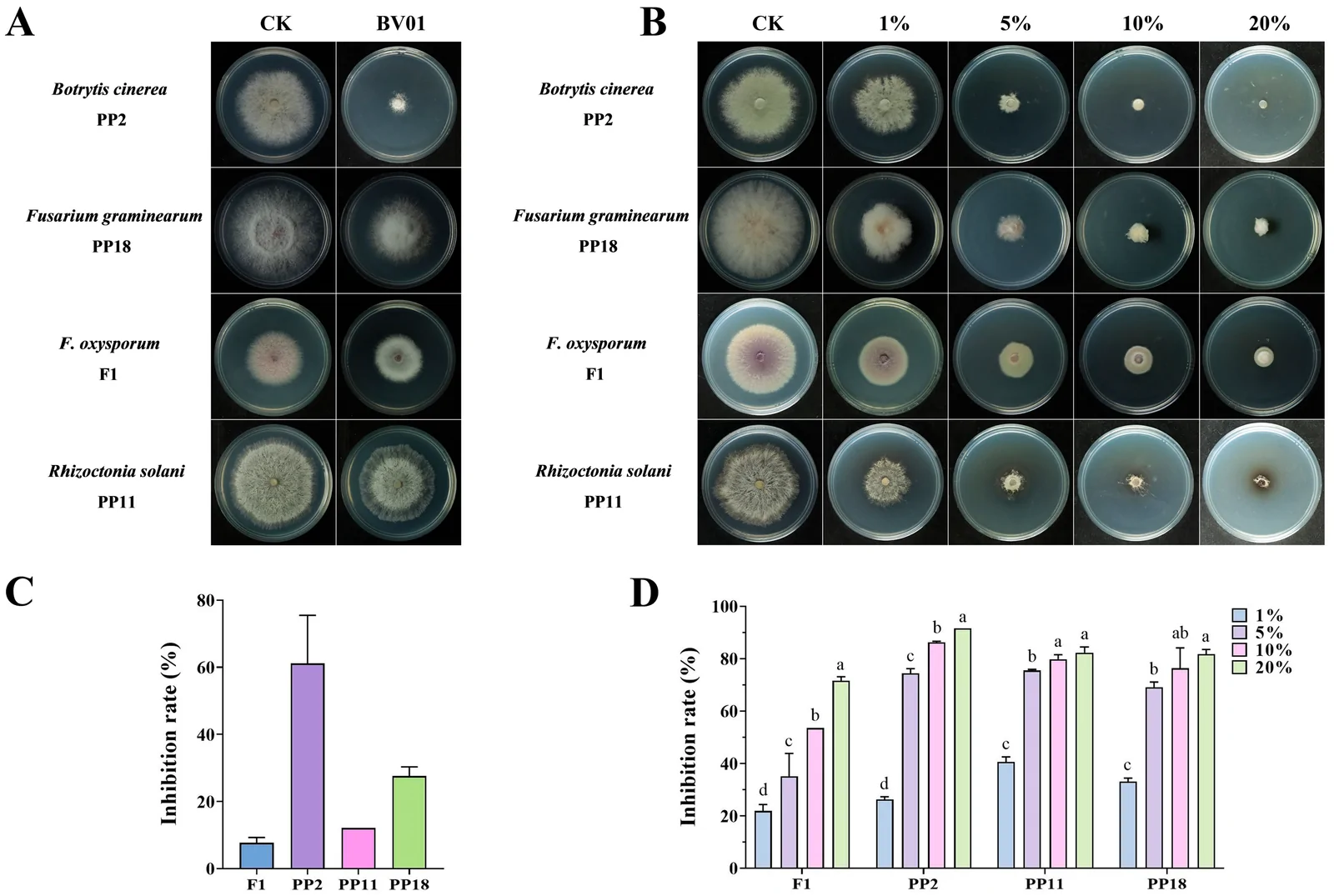
Antifungal activity of BV01 against four phytopathogens. (**A**) Mycelial growth inhibition by VOCs. (**B**) Mycelial growth inhibition by cell-free fermentation filtrate at different concentrations (1%, 5%, 10%, 20%, *v*/*v*). (**C**) Inhibition rates of the VOCs against the four phytopathogens. (**D**) Inhibition rates of cell-free fermentation filtrate against the four phytopathogens. Values are presented as means ± standard errors (SEs). Different letters indicate significant differences (Duncan’s multiple range test, *p* < 0.05).

The inhibitory activity of VOCs produced by strain BV01 against the four phytopathogens was evaluated using the sealed-plate method. Compared with control, the VOCs of BV01 significantly suppressed mycelial growth of all tested pathogens, with inhibition rates ranging from 7.7% to 61.1%. The strongest inhibition was observed against *B. cinerea* PP2 (61.1 ± 8.3%), whereas the weakest was recorded against *F. oxysporum* F1 (7.7 ± 0.92%) (Figure 1A,C).

Additionally, the antifungal activity of the sterile fermentation broth was assessed. The cell-free filtrate of BV01 also exhibited strong inhibitory effects against all pathogens, with inhibition rates ranging from 23.1% to 91.5%. Moreover, the mycelial growth rate of each pathogen on PDA decreased progressively with increasing concentrations of the BV01 sterile filtrate (Figure 1B,D).

### 3.2. Taxonomic Confirmation of BV01

**Figure 2:**
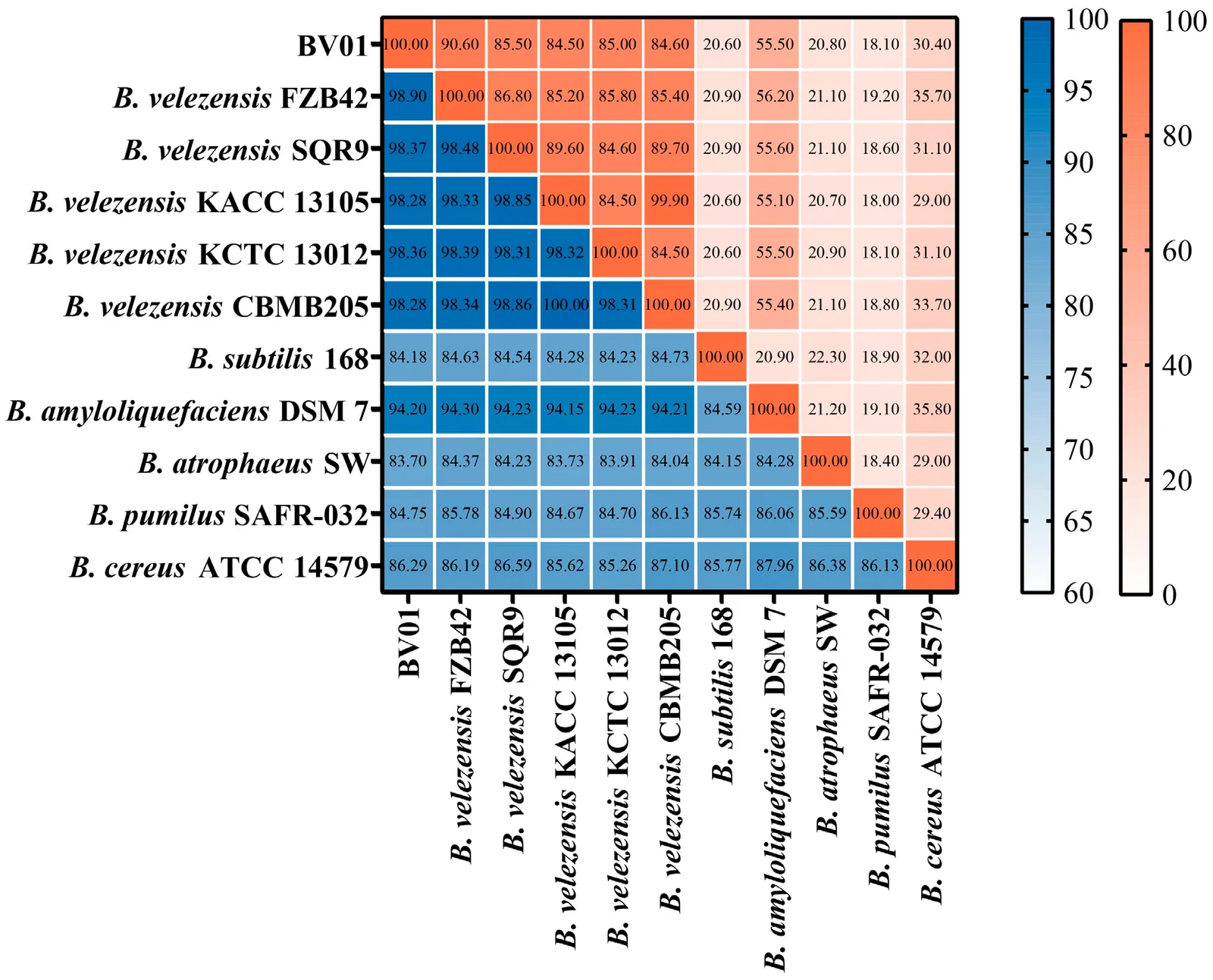
Heatmap showing the pairwise ANI and DDH values between BV01 and 10 *Bacillus* strains. ANI and DDH values are indicated by blue and orange, respectively.

Pairwise ANI and DDH analyses were conducted between BV01 and ten other *Bacillus* strains. The results showed that the ANI values of BV01 against five *B. velezensis* strains exceeded the 95% threshold for species delineation, and the corresponding DDH values also surpassed the 70% cutoff (Figure 2).

Specifically, BV01 showed ANI values of 98.9%, 98.37%, 98.28%, 98.36%, and 98.28% compared to the *B. velezensis* strains FZB42, SQR9, KACC 13105, KCTC 13012, and CBMB205, respectively, with corresponding DDH values reached 90.60%, 85.5%, 84.5%, 85%, and 84.6%. In contrast, comparisons with the strains of other *Bacillus* species yielded values below the species delineation cutoffs, which confirmed that BV01 belongs to *B. velezensis*.

### 3.3. Genomic Characteristics of BV01

**Figure 3:**
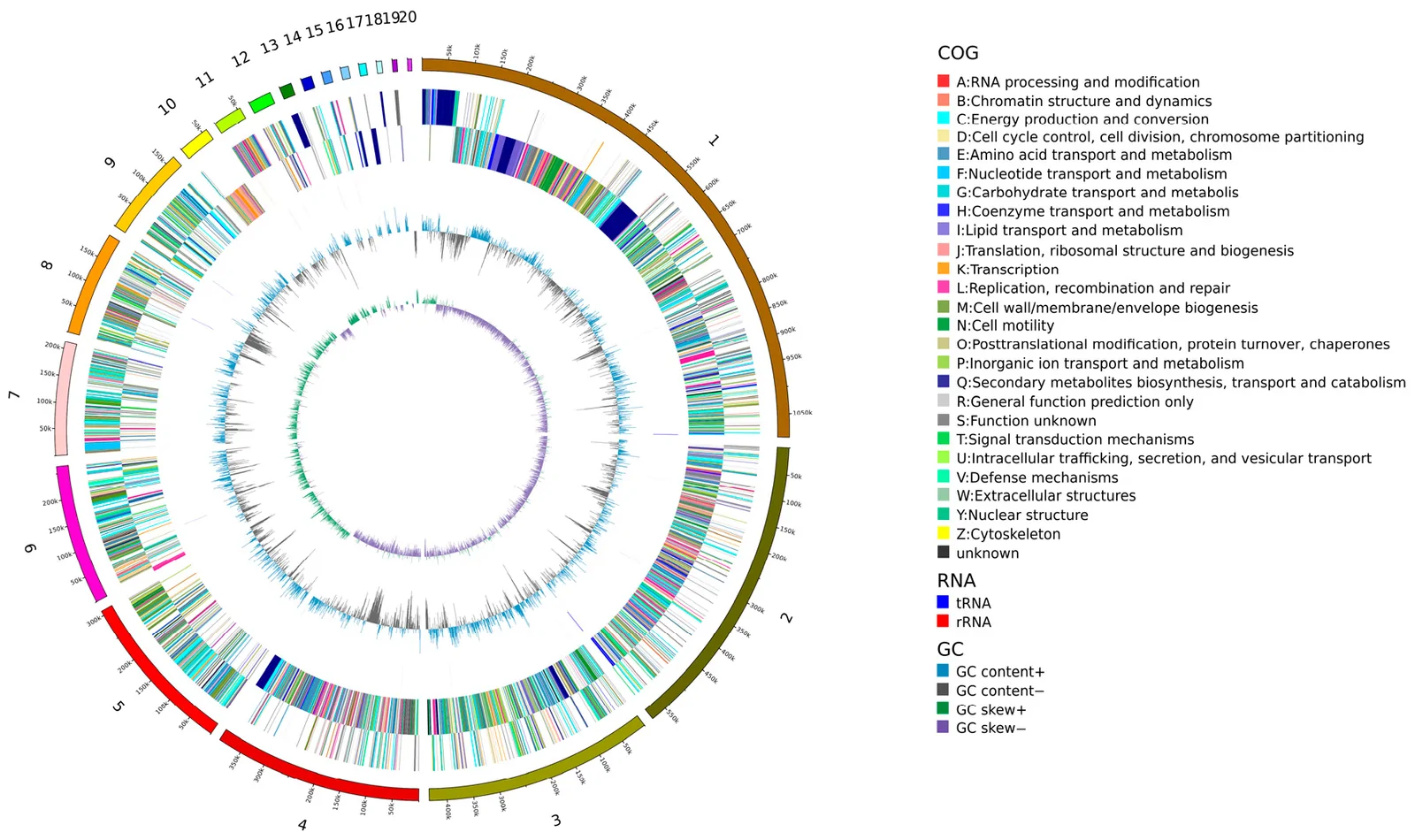
Circular visualization of the top 20 scaffolds (>5000 bp) from the BV01 draft genome. The outer ring displays these scaffolds in order of decreasing size. The concentric rings, from outermost to innermost, display the following features: genomic coordinates, CDSs on the forward-strand, CDSs on the reverse strand, tRNA and rRNA genes, GC content, and GC-skew.

Strain BV01 was sequenced on the Illumina NovaSeq platform using paired-end reads. Sequencing generated a total of 9,365,484 high-quality (HQ) reads, yielding 1,411,960,370 bp of HQ data. The percentages of HQ reads and HQ data were 99.3% and 99.14%, respectively. The estimated sequencing depth was approximately 357×. The Q20 and Q30 values were 98.88% and 96.75%, indicating high overall sequencing quality and suitability for downstream assembly and gene cluster analysis. Assembly of the high-quality reads yielded a draft genome assembly comprising 30 scaffolds, constructed from 32 contigs (Figure 3). The total assembly length was 3,950,525 bp with a GC content of 46.4%. The scaffold N50 was 433,749 bp, and the contig N50 was 395,255 bp. The longest scaffold and contig were 1,094,506 bp and 726,937 bp, respectively. Completeness and contamination were assessed as 99.63% and 0, respectively. The assembly statistics of the BV01 draft genome are summarized in Table 2.

Gene prediction via GeneMarkS v4.32 identified 3,987 protein-coding sequences (CDSs), which collectively accounted for 88.2% of the whole genome. The genome also contains 72 tRNA genes, along with two copies of 5S rRNA, and one copy each of 16S and 23S rRNA (Table 3). As shown in Table 3, the reduced copy numbers of 16S rRNA and 23S rRNA result from the collapse of identical ribosomal repeats during Illumina NovaSeq short-read assembly.

### 3.4. Functional Genome Annotation and Metabolic Profiling of BV01

**Figure 4:**
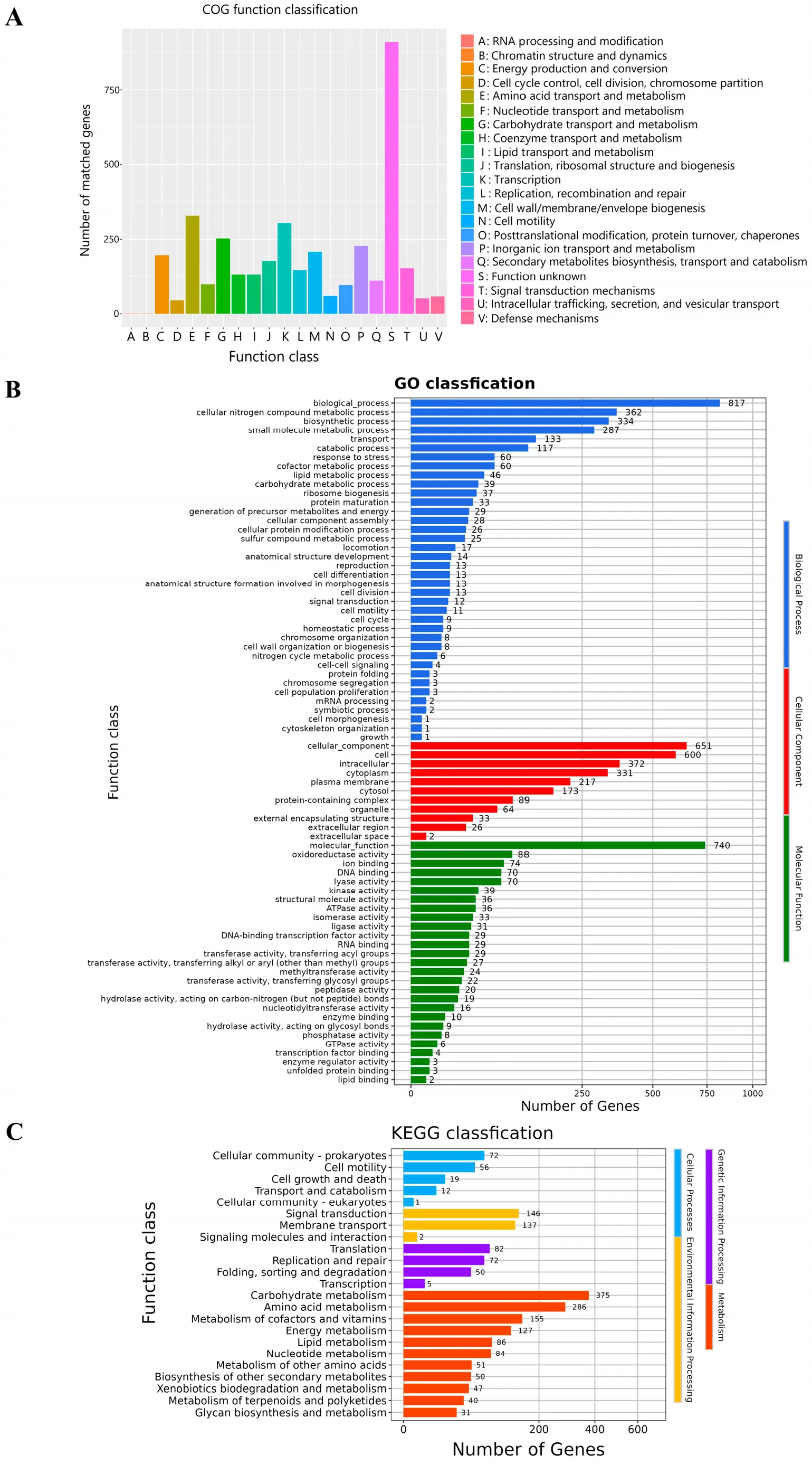
Functional annotation of BV01 based on (**A**) eggNOG, (**B**) GO, and (**C**) KEGG databases.

Functional annotation against the eggNOG database categorized 3689 genes (92.5% of predicted genes) in BV01 into 21 functional classes (Figure 4A). Amino acid transport and metabolism emerged as the most abundant group (329 genes, 8.92% of annotated proteins), followed by transcription (304 genes, 8.24%), and carbohydrate transport and metabolism (253 genes, 6.86%). Additionally, 197, 131, 131, and 110 genes were related to energy production and conversion; coenzyme transport and metabolism; lipid transport and metabolism; and secondary metabolite biosynthesis, transport, and catabolism, respectively.

GO annotation categorized the predicted genes of BV01 into three functional groups: cellular component, molecular function, and biological process (Figure 4B). Within the cellular component category, 651 genes were annotated, mostly encoding products localized to subcellular structures. For molecular function, 740 genes were assigned, predominantly involved in catalytic activities. The biological process category encompassed 817 genes, largely participating in intricate intracellular metabolic networks. In connection with biological process, genes associated with cellular nitrogen compound metabolic process were the most abundant (44.31%), followed by biosynthetic process (40.88%), small molecule metabolic process (35.13%), and transport (16.28%).

KEGG annotation assigned 1986 genes to four major functional categories: metabolism, cellular processes, genetic information processing, and environmental information processing (Figure 4C). Among them, metabolism-related genes constituted the largest fraction, followed by those involved in environmental and genetic information processing. In relation to the metabolism category, carbohydrate metabolism was the most predominant (375 genes), followed by amino acid metabolism (286), cofactor and vitamin metabolism (155), energy metabolism (127), and terpenoid and polyketide metabolism (40). In the environmental information processing category, 146 genes were linked to signal transduction, and 137 to membrane transport. Overall, the KEGG annotation results emphasized carbohydrate and amino acid metabolism as the dominant pathways, underscoring its efficient nutrient utilization and biosynthetic capacity, consistent with its high proliferative activity. Notably, the presence of 40 genes involved in terpenoid and polyketide metabolism (3% metabolic genes) suggests a potential for antimicrobial production, warranting further functional validation to confirm its role in pathogen inhibition.

### 3.5. Genome CAZymes Annotation of BV01

**Figure 5:**
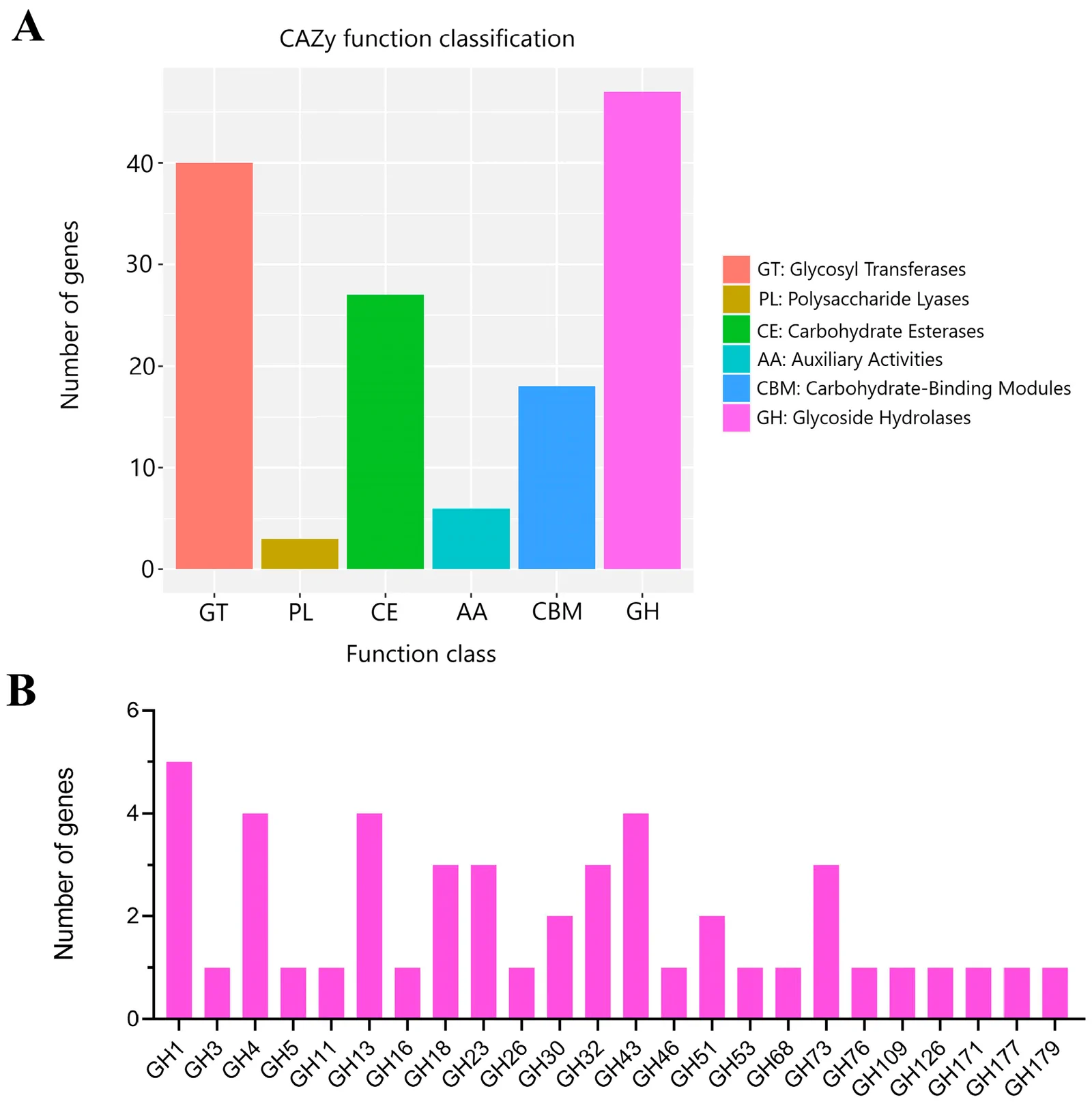
Functional annotation of BV01 based on CAZy database. (**A**) Distribution of gene counts across CAZy families. (**B**) Functional classification of GH families according to the CAZy database.

Genome-wide CAZy annotation predicted that BV01 possesses 141 genes encoding carbohydrate-active enzymes (CAZymes), accounting for 3.54% of the total predicted genes. Based on the sequences and structural features, these genes were classified into six major categories (Figure 5A): glycoside hydrolases (GHs) constituted the largest group (33.33%), followed by glycosyl transferases (28.37%), carbohydrate esterases (19.15%), carbohydrate-binding modules (12.77%), auxiliary activities (4.26%), and polysaccharide lyases (2.13%). Further profiling of the GH family reveals a diverse hydrolase, including cellulase (GH5), chitinase (GH18), chitosanase (GH46), and lysozyme (GH23) (Figure 5B).

### 3.6. Secondary Metabolite Biosynthetic Gene Cluster Prediction and Analysis of BV01

AntiSMASH analysis revealed 15 biosynthetic gene clusters (BGCs) in BV01, including eight known and seven orphan clusters (Table 4). Among the known ones, four BGCs, namely bacillaene, macrolactin H, bacillibactin, and bacilysin, exhibited 100% KnownClusterBlast similarity to database entries, while an additional cluster showed 80% similarity to the fengycin synthesis gene cluster. These similarity values represent the proportion of genes in the reference cluster that have matches in the query cluster, and these high-coverage matches suggest that the strain may have the genetic capacity to produce antimicrobial compounds. The coexistence of both characterized and orphan BGCs provides a solid genetic foundation for the biosynthetic capacity of BV01, which may validate its observed antagonistic activity against phytopathogens.

### 3.7. Comparative Genomics Analysis of BV01 and Related Strains

Strain BV01 was compared genomically with four *Bacillus* strains used in biocontrol studies: *B. velezensis* FZB42 and SQR9, *B. amyloliquefaciens* DSM 7, and *B. subtilis* 168 (Table 5). All the five strains had similar genome sizes and CDS counts. The GC content of BV01 (46.4%) obviously matched those of the other three *Bacillus* strains (46.5%, 46.1%, and 46.1%), but was substantially higher than that of *B. subtilis* 168 (43.5%).

**Figure 6:**
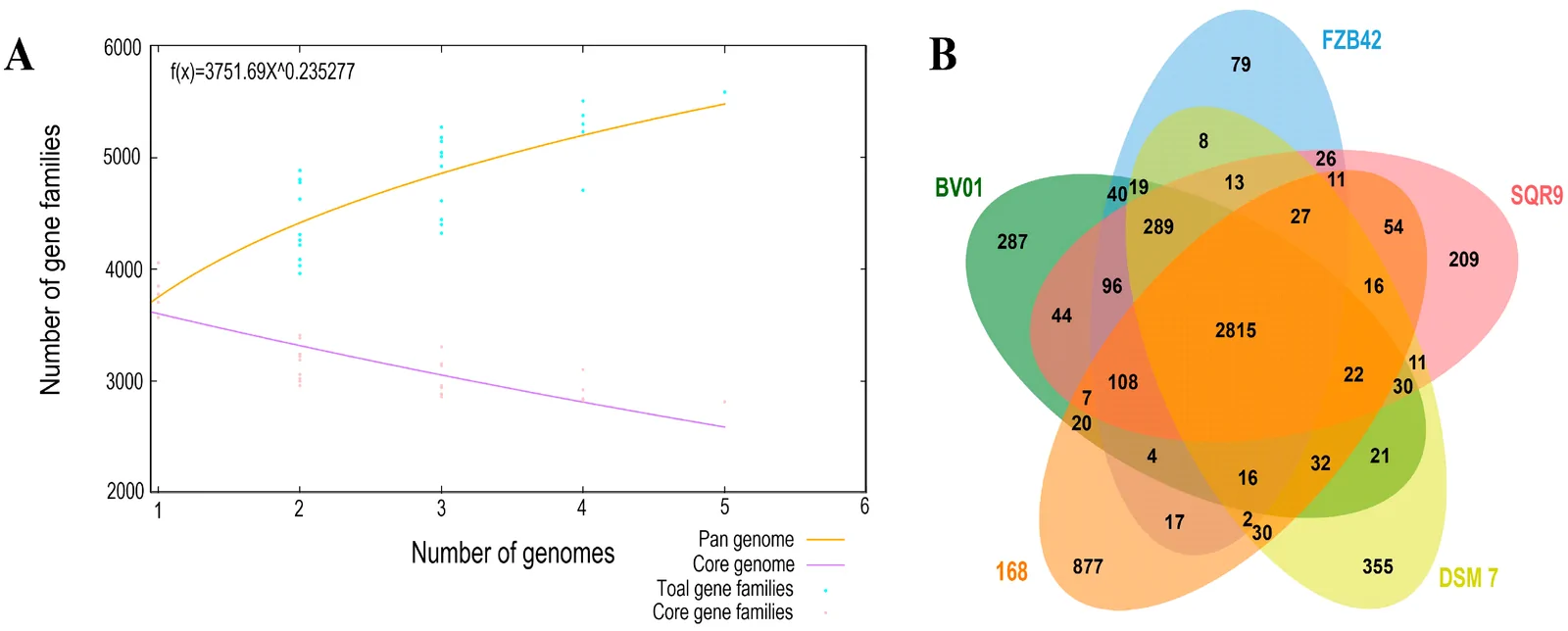
Comparative genomics of BV01. (**A**) Core- and pan-genome plots. (**B**) Venn diagram of gene cluster overlap among BV01 and four reference *Bacillus* strains. Non-overlapping regions indicate gene clusters unique to each strain, whereas overlapping regions indicate gene clusters shared by the corresponding strains. The central region represents gene clusters shared by all five strains.

Pan-genome analysis showed that as the number of strains increased, the pan-genome expanded following a power-law growth trend, whereas the core genome decayed exponentially. The fitted equation yielded a b value of 0.235277 (Figure 6A). According to the Heaps’ law criterion (b < 1), the pan-genome remains open. However, the relatively low b value suggests a slow expansion rate, implying that although the pan-genome has not yet reached a full closed state, the rate of novel gene discovery has declined substantially, and the gene repertoire may be approaching a practical plateau within the sampled taxonomic range. The analysis identified 3850 gene clusters in BV01, which is somewhat higher compared with 3570 in FZB42, 3778 in SQR9, 3706 in DSM7, and 4058 in 168. A total of 2815 core genes were shared among BV01 and the other four reference strains of *Bacillus* (Figure 6B), suggesting their close evolutionary relationships. In addition, BV01 harbored 287 unique genes, which may confer its distinct functional traits.

## 4. Discussion

Using biocontrol microorganisms to manage plant fungal diseases represents a promising and sustainable plant protection strategy [56]. *Bacillus* species possess various antagonistic mechanisms that effectively inhibit a broad spectrum of phytopathogens, and thus become excellent candidates for biocontrol [21,57]. Several species, including *B. amyloliquefaciens*, *B. pumilus*, *B. siamensis*, *B. subtilis*, and *B. velezensis*, have been frequently applied against various plant pathogens [31,58,59,60,61]. Numerous studies have confirmed the strong antagonistic activities of diverse *B. velezensis* strains. For example, strain K01 exhibits potent inhibitory activity against 12 pathogenic fungi [62]; fermentation broth of strain 20507 significantly suppresses mycelial growth of *F. graminearum* [63]; and VOCs produced by strain BF01 considerably inhibit mycelial growth of *Botrytis cinerea*, *F. oxysporum*, and *Colletotrichum gloeosporioides* [64].

Although accumulating evidence suggests the broad-spectrum antagonistic potential of *B. velezensis*, comprehensive research remains scarce, particularly regarding concentration-dependent antifungal activity of fermentation broths from a single strain, inhibitory effects of VOCs, and the genetic basis underlying these traits. To address the gaps, the present study focused on *B. velezensis* BV01 and demonstrated its potent antagonistic activity against *B. cinerea*, *F. graminearum*, *F. oxysporum*, and *Rhizoctonia solani*. Notably, fermentation broths inhibited mycelial growth in a dose-dependent manner, and BV01-derived VOCs also suppressed the growth of all the four pathogens, with the strongest inhibition observed to *B. cinerea*.

The biocontrol activities of *Bacillus* species are largely attributable to their diverse antimicrobial metabolites, including polyketides and non-ribosomal lipopeptides [23,26,65]. Genome analysis of BV01 revealed 15 BGCs, and some of them are responsible for the productions of known antimicrobial substances, thus providing a genetic foundation for its biocontrol potential. Among them, bacillaene and macrolactin H are polyketides with antibiotic properties primarily targeting bacterial phytopathogens [66]. Bacillibactin, a catechol-type siderophore, enables BV01 to chelate limited iron in the environment, thereby suppressing the growth of the competing pathogens [2]. Bacilysin inhibits glucosamine-6-phosphate synthase, interfering with the synthesis of essential cell wall components of pathogens and exerting both antifungal and antibacterial effects [67].

A gene cluster showing 80% KnownClusterBlast similarity to the known fengycin synthetase gene cluster was identified in the BV01 genome. Fengycin displays strong antagonistic activity against filamentous fungi primarily through interacting with membrane sterols and phospholipids, and thereby disrupting the membrane integrity and permeability of pathogens [68]. The presence of this cluster positions it as a prime candidate responsible for the observed antifungal activity. Its divergence from the known cluster may imply structural or synthetic variations in its products, potentially modulating its bioactivity. Thus, it merits further functional investigation as a potential determinant of BV01’s antifungal activity.

Compared with the reference sequences in the Minimum Information about a Biosynthetic Gene Cluster (MIBiG) database, BV01 possesses BGCs for bacillaene, macrolactin H, bacillibactin, and bacilysin (100% KnownClusterBlast similarity), whereas its clusters for difficidin and surfactin display relatively low similarity. These different similarities suggest that BV01 may employ a unique biocontrol mechanism or exhibit a distinct antimicrobial spectrum. Collectively, the diverse secondary-metabolite BGCs in the BV01 genome constitute a robust genetic basis for its potent biocontrol activity. Although the BGCs identified in BV01 provide a genomic basis for its biocontrol potential, their actual contributions remain to be experimentally confirmed. Future work will therefore include liquid chromatography–mass spectrometry (LC-MS) analysis to verify whether the putative metabolites encoded by these BGCs, such as bacillaene, macrolactin H, bacillibactin, bacilysin, and fengycin, are produced, thereby strengthening the mechanistic link between genomic potential and the observed biocontrol phenotypes.

Many *Bacillus* species suppress phytopathogens by secreting of cell-wall-degrading enzymes, such as proteases, cellulases, glucanases, and chitinases, which hydrolyze key structural components of fungal cell walls, thus inhibiting pathogen growth and reducing pathogenicity [25,69,70]. In BV01, this ability is supported at both the genetic and phenotypic levels: its genome harbors multiple genes encoding such enzymes, and extracellular production of proteases, cellulases, and β-1,3-glucanases has been demonstrated in our previous study [36], which likely contributes to its antagonistic activity against phytopathogenic fungi.

## 5. Conclusions

In summary, the fermentation broth of *B. velezensis* BV01 exhibited concentration-dependent inhibition of mycelial growth against *Botrytis cinerea*, *Fusarium graminearum*, *F. oxysporum*, and *Rhizoctonia solani*, with inhibition rates ranging from 23.1% to 91.5% across tested concentrations (1–20%). Its VOCs also suppressed the growth of the pathogens, with species-specific efficacy. Genome analyses identified multiple BGCs encoding antimicrobial metabolites, along with genes for various hydrolytic enzymes (e.g., proteases, cellulases, and glucanases), providing a molecular basis for its biocontrol activity. Collectively, these findings indicate that BV01 holds considerable promise as an effective multifaceted biocontrol agent.

## Figures and Tables

**Table 1:** *Bacillus* strains used for ANI and DDH analyses.

Strain	NCBI RefSeq Accession Number
*Bacillus velezensis* FZB42	GCF_000015785.2
*B. velezensis* SQR9	GCF_000685725.1
*B. velezensis* KACC 13105	GCF_000960265.2
*B. velezensis* KCTC 13012	GCF_001267695.1
*B. velezensis* CBMB205	GCF_002117165.1
*B. subtilis* 168	GCF_000009045.1
*B. amyloliquefaciens* DSM7	GCF_000196735.1
*B. atrophaeus* SW	GCF_039519175.1
*B. pumilus* SAFR-032	GCF_000017885.4
*B. cereus* ATCC 14579	GCF_046524075.1

**Table 2:** Assembly statistics of the BV01 draft genome.

Item	Value
Raw data	1.42 Gb
Clean data	1.41 Gb
Q20	98.88%
Q30	96.75%
Coverage depth	357×
Total assembly length	3,950,525 bp
GC content	46.4%
Number of scaffolds	30
Number of contigs	32
Scaffold N50	433,749 bp
Contig N50	395,255 bp
Longest scaffold	1,094,506 bp
Longest contig	726,937 bp
Completeness	99.63%
Contamination	0

**Table 3:** Genome annotation statistics of the BV01 draft genome.

General Features	*B. velezensis* BV01
Protein-coding sequences (CDSs)	3987
Longest CDS length (bp)	16,170
Average CDS length (bp)	874
CDS/genome (%)	88.2%
Number of tRNA	72
Number of 5S rRNA	2
Number of 16S rRNA	1
Number of 23S rRNA	1
Number of ncRNA	84
Number of minisatellite DNA	90
Number of microsatellite DNA	1

**Table 4:** Biosynthetic gene clusters of BV01 predicted by AntiSMASH.

Cluster	Type	Location	Most Similar Known Cluster	Similarity
Cluster 1	Nonribosomal peptide synthetase (NRPS), betalactone, transAT-PKS	Scaffold_1: 1-87944	fengycin	80%
Cluster 2	transAT-PKS, NRPS, T3PKS	Scaffold_1: 150037-260142	bacillaene	100%
Cluster 3	transAT-PKS	Scaffold_1: 479998-568225	macrolactin H	100%
Cluster 4	terpene	Scaffold_1: 881374-902114	N/A	N/A
Cluster 5	PKS-like	Scaffold_1: 987910-1029154	N/A	N/A
Cluster 6	NRP-metallophore, NRPS, RiPP-like	Scaffold_3: 77001-128789	bacillibactin	100%
Cluster 7	terpene	Scaffold_4: 87246-109129	N/A	N/A
Cluster 8	T3PKS	Scaffold_4: 192786-233886	N/A	N/A
Cluster 9	transAT-PKS-like	Scaffold_4: 349473-395255	difficidin	53%
Cluster 10	other	Scaffold_5: 38294-79712	bacilysin	100%
Cluster 11	transAT-PKS-like	Scaffold_13: 1-22944	difficidin	26%
Cluster 12	NRPS	Scaffold_16: 1-15243	surfactin	39%
Cluster 13	NRPS	Scaffold_17: 1-13863	N/A	N/A
Cluster 14	NRPS	Scaffold_18: 1-10221	N/A	N/A
Cluster 15	NRPS	Scaffold_21: 1-7674	N/A	N/A

N/A: No obvious similar known cluster was detected by antiSMASH.

**Table 5:** Genomic features of *B. velezensis* BV01 compared with four reference *Bacillus* strains.

Features	BV01	FZB42	SQR9	DSM 7	168
Genome size (bp)	3,950,525	3,918,600	4,117,023	3,980,200	4,215,606
GC content (mol%)	46.4	46.5	46.1	46.1	43.5
Protein-coding sequences	3987	3693	4078	3921	4237
Average CDS size (bp)	874	933	916	888	895
Percent of coding region (%)	88.2	88.0	89.0	87.0	87.2
Numbers of tRNAs	72	88	72	93	86

## Data Availability

The raw sequencing data have been deposited in the NCBI Sequence Read Archive (SRA) under BioProject accession number PRJNA1530769 (BioSample: SAMN63192874), and the assembled draft genome has been deposited in GenBank under accession number JCDZBJ010000000.
